# Factors Governing B Cell Recognition of Autoantigen and Function in Type 1 Diabetes

**DOI:** 10.3390/antib13020027

**Published:** 2024-04-01

**Authors:** Lindsay E. Bass, Rachel H. Bonami

**Affiliations:** 1 Department of Pathology, Microbiology and Immunology, Vanderbilt University Medical Center, Nashville, TN 37232, USA; lindsay.e.bass@vanderbilt.edu; 2 Department of Medicine, Division of Rheumatology and Immunology, Vanderbilt University Medical Center, Nashville, TN 37232, USA; 3 Vanderbilt Center for Immunobiology, Vanderbilt University Medical Center, Nashville, TN 37232, USA; 4 Vanderbilt Institute for Infection, Immunology and Inflammation, Vanderbilt University Medical Center, Nashville, TN 37232, USA

**Keywords:** type 1 diabetes, B lymphocytes, B cell receptor, T lymphocytes, insulin, autoantigen

## Abstract

Islet autoantibodies predict type 1 diabetes (T1D) but can be transient in murine and human T1D and are not thought to be directly pathogenic. Rather, these autoantibodies signal B cell activity as antigen-presenting cells (APCs) that present islet autoantigen to diabetogenic T cells to promote T1D pathogenesis. Disrupting B cell APC function prevents T1D in mouse models and has shown promise in clinical trials. Autoantigen-specific B cells thus hold potential as sophisticated T1D biomarkers and therapeutic targets. B cell receptor (BCR) somatic hypermutation is a mechanism by which B cells increase affinity for islet autoantigen. High-affinity B and T cell responses are selected in protective immune responses, but immune tolerance mechanisms are known to censor highly autoreactive clones in autoimmunity, including T1D. Thus, different selection rules often apply to autoimmune disease settings (as opposed to protective host immunity), where different autoantigen affinity ceilings are tolerated based on variations in host genetics and environment. This review will explore what is currently known regarding B cell signaling, selection, and interaction with T cells to promote T1D pathogenesis.

## 1. Introduction

Type 1 diabetes (T1D) is a chronic autoimmune disease that results in T cell-mediated destruction of pancreatic beta cells and impaired insulin production [1,2,3]. Islet autoantibodies are predictive T1D biomarkers and can be detected months to years before clinical diagnosis [4]. In some autoimmune diseases, such as systemic lupus erythematosus or rheumatoid arthritis, autoantibodies directly mediate tissue damage; this was in part deduced from experiments in animal models in which passive transfer of serum autoantibodies was sufficient to cause disease-related pathology [5,6]. In contrast, passive transfer of serum autoantibodies was not sufficient to cause beta-cell damage in the non-obese diabetic (NOD) mouse model of T1D [7]. B cell-deficient NOD mice were protected from diabetes, which was ascribed to their function as antigen-presenting cells (APCs) [7,8]. Here, we will review how islet-reactive B cells develop and function to promote T1D, and we will provide an overview of how a growing understanding of T1D immunology is being tapped to develop new therapies for T1D.

## 2. T1D Prevalence, Staging, and Clinical Challenges

As of 2020, the global prevalence of T1D is estimated at 5.9 cases per 10,000 people [9] and is expected to double by 2040 [10]. The economic burden of T1D is estimated to have an extra lifetime cost of USD 813 billion for a cohort of ~1.6 million T1D patients compared to non-T1D individuals [11]. Those with T1D have mortality rates that are two to eighteen times higher than would be expected in their respective countries [12,13,14]. Improved immunotherapies that prevent T1D onset and disease progression could thus offer significant quality-of-life and economic benefit.

Major hurdles to successful immunotherapy development include research limitations and heterogeneous human T1D etiopathogenesis. One major hurdle in T1D research is the lack of translation of therapeutic success observed in the NOD mouse model to humans, due to both known and unknown differences in disease pathogenesis, as reviewed previously [15]. For example, 80% of female and 20% of male NOD mice develop T1D [16]. This strong female bias is not observed in human T1D [17,18,19], as is seen in other autoimmune diseases including systemic lupus erythematosus [20]. In addition, differences in expression and polymorphisms in major histocompatibility complex (MHC) class II molecules, which confer disease risk, may contribute to discrepancies in immunotherapeutic responses between NOD mice and humans [15]. To support translational studies in human T1D, major efforts were undertaken to establish access to human biospecimens, particularly during the pre-clinical stages of T1D. These efforts included (but were not limited to) the establishment of the T1D research consortium, Type 1 Diabetes TrialNet, and the T1Detect screening program launched by the Juvenile Diabetes Research Foundation (JDRF) [21,22]. Peripheral blood is accessible and amenable to longitudinal sampling, with the caveat that immunological findings in the peripheral blood may not align with pathologic responses in pancreatic tissue. To provide access to key T1D tissues, the Network for Pancreatic Organ Donors with Diabetes (nPOD) was developed in 2007 by the JDRF to obtain tissues from cadaveric organ donors (including individuals with T1D) to enable the direct study of immune cells in T1D-relevant tissues [23].

Positivity for two or more islet autoantibodies against insulin (IAA), glutamic acid decarboxylase 65 (GAD65), tyrosine phosphatase-related islet antigen-2 (IA-2), islet cell autoantigen (ICA), or zinc transporter 8 (ZnT8) confers a >80% risk of developing T1D within 20 years and is used together with glycemia data for the classification of T1D into disease stages [3,4,24]. In addition to islet autoantibody positivity, Stage 1 is defined by normoglycemia, as established by oral glucose tolerance test results outlined by Insel et al. [4]. Stage 2 is defined by impaired glucose tolerance [4]. Finally, Stage 3 is marked by overt dysglycemia and/or the presentation of clinical symptoms, including excessive thirst, weight loss, fatigue, and appetite and urination changes, and results in clinical diagnosis [4]. T1D typically requires lifelong insulin hormone replacement therapy via injection. Even with insulin therapy and adequate clinical follow-up, the majority of T1D patients struggle to achieve target glycemic control [25]. T1D individuals are at an increased risk of cardiovascular disease, renal disease, and mortality [12,13,14]. Major research efforts have thus focused on determining the immune drivers of T1D immunopathology to uncover new biomarkers and therapeutic targets.

Heterogeneity in T1D onset and response to therapy is a major challenge for immunotherapy development and clinical use. Although highly debated, the classification of T1D endotypes or disease subsets, defined by differences in clinical, genetic, and immunological factors, could help address the complexity of the disease and inform treatment strategy. Two T1D endotypes have been defined; Endotype 1 is defined by early diagnosis, extensive beta-cell destruction, aggressive insulitis of both B and T cells [26], and aberrant proinsulin processing [27], whereas Endotype 2 is defined by later diagnosis, retention of residual insulin-containing islets, fewer infiltrating B and T cells, and normal proinsulin processing [27]. The identification and validation of additional endotypes could help refine clinical trial inclusion criteria to focus on people with the specific T1D endotypes who are most likely to benefit from a given therapy (increasing the likelihood of reaching primary clinical trial endpoints) and could ultimately support personalized medicine for T1D. Additional research is needed to fully reach these goals.

Insufficiency of early prognostic and predictive biomarkers reflecting the heterogeneity of T1D further hinders T1D immunotherapy development. Although well-established as T1D biomarkers, autoantibodies are often transient [28,29]. Further, studies in VH125Tg.NOD mice show the potential for aggressive islet-reactive B lymphocyte responses in the absence of detec insulin autoantibodies, due to immune tolerance blockade of antibody production [30,31]. Beta-cell-related markers of disease progression, including C-peptide and hemoglobin A1c (HbA1c) levels, require tissue damage to have already occurred. Identification of earlier immunological biomarkers that can differentiate T1D endotypes could thus offer an earlier window to modify intervention, and in the clinical trial setting, better inform risk classification and participant stratification to improve clinical trial development and clinical management in the future.

## 3. Murine B Lymphocytes Present Islet Autoantigens to T Cells in T1D

Leete et al. showed that increased B cell infiltration of islets corresponds with early-onset and more aggressive disease in humans [26], yet human pancreatic tissue is relatively inaccessible. Therefore, much of what we have learned about T and B cell contributions to T1D originated from studies of NOD mice, which share many of the same genetic features as human T1D and spontaneously develop diabetes following autoimmune attack on beta cells [16]. As mentioned above, APC function, rather than autoantibody production, is considered the major mechanism by which B cells contribute to T1D. Table 1 provides an overview of several key NOD mouse models that were used to ascertain B cell contributions to T1D pathogenesis.

In 1996, Serreze et al. showed that B cell-deficient NOD.Igμ mice were protected from T1D [32]. T1D susceptibility and GAD-specific T cell responses were restored in NOD.Igμ mice reconstituted with syngeneic bone marrow and NOD B lymphocytes but not those with syngeneic bone marrow only [7]. NOD mice with B cell-specific deficiency in MHC class II molecule I-Ag7 are protected from T1D [33]. B-cell-depleted CD4+ NOD T cells were unable to divide in response to soluble anti-CD3 and anti-CD28 stimulation, in contrast to B6 CD4+ T cells, which underwent successive rounds of division during the culture period. APC-depleted B6 and NOD CD4+ T cells stimulated with plate-bound CD3 underwent cell division, suggesting that impaired NOD CD4+ T cell division is not a T cell-intrinsic impairment but instead due to dependence on B cell co-stimulation [43]. Interestingly, CD4+ T cell division in response to soluble anti-CD3 and anti-CD28 was not impaired in B cell-deficient NOD.Igμ mice splenocytes as observed in the anti-B220 depletion model above [44]. Greeley et al. hypothesized that B cells may be critical for optimal T cell activation in the lymph node specifically and indeed found impaired CD4+ T cell proliferation in response to soluble anti-CD3 and anti-CD28 stimulation compared to wildtype NOD mice [44]. This degree of impairment was not observed in B cell-deficient non-autoimmune B6 mice [44]. Silveira et al. showed that the introduction of a transgenic B cell receptor (BCR) specific for the disease-irrelevant antigen, hen egg lysozyme (HEL), in the NOD.Igμ mouse delayed T1D development and prevented T cell proliferation [8]. These findings suggest that BCR-mediated capture of beta-cell antigen accounts for the critical B cell antigen presentation in NOD mice [8]. This antigen presentation role is further supported by findings in insulin-specific B and T cell NOD models [45,46]. It is postulated that B cells can act as APCs to stimulate CD4+ T cells, which in turn can release cytokines that promote cytotoxic CD8+ T cell-mediated destruction in the pancreas (reviewed in [47,48,49]). Egia-Mendikute et al. show that autoreactive B cells shape T cell phenotype in an antigen-specific, transgenic NOD mouse model [50].

Transgenic VH125Tg.NOD mice, which harbor an increased percentage of insulin-specific B cells (1–2% of the total repertoire detectable by flow cytometry), show increased diabetes incidence compared to WT.NOD mice [34]. In contrast, transgenic NOD mice that lack insulin-binding B cells (VH281Tg.NOD) are protected from T1D [34]. Although the VH125Tg.NOD mouse is an effective model to investigate diabetogenic B cells, the BCR from which the transgene was designed originated from BALB/c mice immunized with foreign insulins, and thus may not represent natural autoimmune responses.

Leeth et al. generated and characterized a transgenic NOD mouse model derived from naturally occurring, islet-infiltrating, peripherin-autoreactive B cells, designated NOD-PerIg [38]. Peripherin is expressed widely in the neuronal cells of the central and peripheral nervous system but is also expressed in the peri-insular areas of postnatal mice pancreata [51,52,53]. Peripherin-autoreactive B cells infiltrate the pancreas, acquire an activated phenotype, and exhibit increased MHC class I and II expression [38]. In NOD.scid.PerIg mice, which harbor peripherin-autoreactive B cells but are T cell deficient, engraftment with NOD T cells without previous opportunity to interact with pathogenic B cells led to increased T1D development compared to B and T cell-deficient NOD.SCID mice [38]. Together, transgenic mouse studies provide strong rationale for autoantigen-specific B cells as contributors to T1D pathogenesis.

## 4. BCR Signaling and B Cell Tolerance Break in T1D

BCR signaling is required for B cell maturation and survival [54,55,56,57,58,59]. B cells arrest at the pro B cell stage in µMT mice, which lack the IgM transmembrane domain necessary to support BCR expression on the cell surface [60]. µMT B cell maturation is however restored by the provision of intracellular Igα/Igβ complexes with intact immunoreceptor tyrosine-based activation motifs (ITAMs) that associate with the plasma membrane, highlighting a role of antigen-independent, tonic BCR signaling in supporting B cell development and maturation in the periphery [61]. During development in the bone marrow, B cells are also tested for reactivity to self-antigens. Using a high-affinity, HEL-specific BCR transgenic mouse model, Hartley et al. showed that when HEL was expressed in membrane form at the cell surface, HEL-specific B cells were eliminated through the immune tolerance mechanism, deletion [62]. Developing B cells can also undergo receptor editing, whereby continued light chain rearrangement can replace an autoreactive light chain with a new light chain that mitigates self-antigen reactivity [63,64]. Despite various central tolerance mechanisms, Wardemann et al. showed that even in healthy humans, up to 75% of newly generated B cells show some degree of autoreactivity, but this decreases to about 20% in mature B cells [65]. This is accomplished through peripheral immune tolerance checkpoints, including deletion (outlined above) and anergy (defined below), which requires BCR distinction between self and non-self. Building on Bretscher and Cohn’s two signal model of lymphocyte activation [66], B cells require initial BCR autoantigen recognition, followed by a second signal from cognate T cell help or innate signaling within a certain period of time to promote B cell survival and differentiation into antibody-secreting cells [67]. The absence of these secondary signals can promote tolerance through mitochondrial dysfunction, cell death, or anergy [67,68,69].

Anergy is an immune tolerance mechanism that limits a B cell’s ability to participate fully in an immune response. Initially described in transgenic HEL-specific B cells that chronically encounter soluble HEL self-antigen, anergy was characterized by reduced surface IgM (but not IgD), reduced proliferation and antibody secretion, and reduced Ca^2+^ flux and tyrosine phosphorylation [70,71,72]. However, this impaired state is reversible, as shown by the functional recovery exhibited in an environment without autoantigen present [73]. In the same HEL-transgenic mouse model, Ubelhart et al. showed that although anergic B cells were rendered unresponsive to soluble antigens, they remained fully responsive to multivalent antigens through IgD engagement [74]. Similar functional impairment to that observed in the HEL model was shown in a p-azophenylarsonate-specific immunoglobulin transgenic mouse, in which B cells become anergic due to cross-reactivity to autoantigen in the bone marrow [75].

Most B cells (>95%) from 125Tg C57BL/6 mice bind insulin but exhibit impaired calcium mobilization, NFATc1 signaling, and proliferation in response to stimulation [30,76,77]. This anergic state is also observed in 125Tg B cells isolated from NOD mice [76], yet the NOD.125Tg B cells maintain the ability to present antigen, migrate to the pancreas, and promote T1D disease [45,78]. BCR-antigen binding promotes antigen internalization required for antigen presentation. Internalized antigen is processed in the endolysosome, and peptides are loaded onto MHC class II molecules. It has been shown that anergic B cells in non-autoimmune mice display impaired endolysosome fusion, yet this phenotype is reversed on an autoimmune background [79]. Anti-insulin B cells upregulate the T cell costimulatory molecule, CD86, relative to non-insulin-binding B cells present in the pancreas of the same NOD.VH125Tg mice; insulin stimulation in vitro can similarly evoke anti-insulin B cell upregulation of CD86 [80]. Anti-insulin T cell clones have been isolated from mice and humans which recognize a specific peptide register derived from the insulin B chain [81,82]. Germinal center B cells, but not follicular B cells, derived from NOD.VH125^SD^ mice are competent to process insulin and present this pathogenic peptide register to drive in vitro proliferation of pathogenic 8F10 anti-insulin T cell clones [46]. Surprisingly, the 116C.NOD mouse, a transgenic mouse derived from an islet-infiltrating B cell, produces clonal B cells with pancreatic islet beta-cell specificity but is protected from diabetes [39]. 116C.NOD B cells display an anergic phenotype, as described by impaired proliferation and calcium flux in response to stimulation; yet, they are able to produce cytokines that can induce Th17-cell differentiation [39]. Carrascal et al. hypothesize that these 116C.NOD B cells protect from T1D disease due to impaired antigen presentation [39].

In the peripheral blood of healthy humans, insulin-binding B cells have been identified in the anergic compartment but disappear from the anergic subset in prediabetic and new-onset T1D individuals [83]. Later studies by Stensland et al. suggested that anergic, insulin-binding B cells in young-onset T1D individuals adopted an activated phenotype poised for antigen presentation to T cells and differentiation into antibody-secreting cells [84]. Therefore, they proposed a model in which insulin-binding B cells can escape tolerance through anergic silencing, but can become activated in an inflammatory setting, to present autoantigens and promote human T1D pathology.

Antigen exposure can modulate BCR signaling strength, as shown by Nur77 upregulation [85]. BCR signaling strength can impact B cell fate and function [86,87]. BCR signaling strength in response to antigen recognition increases with antigen affinity through a phenomenon known as affinity discrimination [88]. B cells can recognize antigens over a range of affinities from 10^6^ to 10^10^ M^−1^, but the mechanism by which affinity discrimination occurs is currently unresolved [88]. Tsourkas et al. propose kinetic proofreading as a predominant mechanism over serial engagement for affinity discrimination [88]. Kinetic proofreading requires receptor modifications that allow for signal induction, including BCR oligomerization and association with other signaling molecules [89,90,91]. To investigate affinity discrimination with bound antigen, Natkanski et al. used a model of immobilized plasma membrane sheets that mimic APC membranes and found that B cells exerted a force on the BCR-antigen complex to extract antigens from the membrane sheets [92]. A low affinity interaction cannot withstand this force, allowing for B cell affinity discrimination [92]. Sensitivity to antigen/BCR binding may differ across B cell subset, as reviewed in the context of differences in germinal center versus naïve B cell responses to BCR ligation [93].

Secondary signals that synergize with B cell signaling include B cell-activating factor (BAFF), CD40L, toll-like receptors (TLRs), and various cytokines. Anti-BAFF mAb administration depleted BAFF and protected NOD mice from disease [94]. BAFF receptor (BAFFR)-Fc treatment inhibited T1D development; B cells remaining after BAFFR-Fc treatment had a diminished capacity to present autoantigens to diabetogenic T cells and exhibited a regulatory phenotype [95]. B cells isolated from T1D individuals with long-standing diabetes showed increased proliferation when stimulated with BAFF in vitro compared to healthy donors, suggesting altered BAFF signaling in T1D [96]. Treatment with anti-CD40L antibody (limiting T cell–APC collaboration) prevented T1D in the NOD mouse when administered prior to the initiation of insulitis [97]. In support of this finding, a CD40-targeted peptide controlled and even reversed diabetes in NOD mice [98]. CD40 is expressed on a variety of APCs and neither of these studies investigated the impact of impaired CD40/L signaling on diabetogenic B cells specifically.

TLRs recognize pathogen- and/or damage-associated molecular patterns and modulate B cell survival, activation, and function [99]. TLR7 recognizes single-stranded RNA, and TLR7 deficiency protected from T1D in NOD mice [42]. Protection was attributed to altered B cell differentiation and function [42]. The total number of B cells was reduced in the bone marrow and peripheral lymphoid tissues of TLR7-deficient mice [42]. The frequencies of marginal zone and regulatory B cells were significantly increased in the TLR7-deficient NOD mice with a corresponding decrease in germinal center and follicular B cells [42]. TLR7-deficient NOD B cells showed impaired autoantibody production and antigen presentation and increased PD-L1 expression [42]. TLR7 is expressed on a variety of immune cells, and decreased macrophage, CD4-CD8- thymocyte, and peripheral CD4+ T cell populations were observed [42]; therefore, B cell-specific TLR-deficient NOD studies are needed to elucidate the direct impact of TLR signaling in B cells on T1D pathogenesis.

Signaling molecules downstream of the BCR have also been studied in T1D. Bruton’s tyrosine kinase (BTK) is recruited to the cell membrane by phosphorylated Syk and phosphorylates phosphatidylinositol-specific phospholipase Cγ2 (PLCγ2) to activate transcription factors NF-kB and NFAT [100,101,102,103,104,105,106]. *Btk* deficiency in NOD mice alters peripheral B cell subsets, impairs B cell proliferation and insulin autoantibody production, and protects against T1D, whereas the introduction of insulin-specific VH125Tg into *Btk*-deficient NOD mice restored diabetes [37]. Anti-insulin B cells are preferentially sensitive to the loss of *Btk*, yet they retain a normal ability to internalize the BCR in response to BCR stimulation [36]. Taken together, these data suggest that BTK is important for anti-insulin B cell selection, rather than pathogenic APC function, in T1D. Replacement of the tyrosine kinase Syk with hyporesponsive Zap-70 in a Syk Zap-70 knock in mouse model resulted in impaired B cell selection, production of anti-insulin autoantibodies, and increased fasting blood glucose levels that may highlight impaired BCR signaling as a risk factor for T1D development [107].

Packard et al. showed that affinity for insulin autoantigen can regulate the induction of tolerance and antigen presentation in NOD B cells [108]. On the C57BL/6 background, transgenic murine B cells with a high-affinity for insulin were more susceptible to tolerance than low-affinity B cells, as measured by downregulation of surface IgM and reduced BCR signaling following stimulation. Low-affinity NOD B cells were less susceptible to anergy compared to those from C57BL/6 mice. In addition, low-affinity NOD B cells were better able to present antigen to antigen-experienced, insulin-specific T cells, as measured by interferon-γ production [108]. This supports the proposed model in which low-affinity, anergic B cells can escape tolerance to play a role in T1D pathology. NOD mice also showed a reduced propensity to evoke the central immune tolerance mechanism, receptor editing, relative to non-autoimmune mice, which incompletely censored anti-insulin B cells in both strains [109]. Thus, immune tolerance defects in autoimmune mice could enhance autoreactive B cell entry and function in the periphery.

## 5. The Impact of Somatic Hypermutation and Affinity Maturation on BCR Autoantigen Recognition in T1D

The BCR is the membrane-bound form of secreted immunoglobulin, which binds antigen to mediate B cell activation via the signaling co-receptors Igα and Igβ. The BCR is comprised of two heavy chains and two light chains, each containing a variable and a constant region. The variable region contributes to the antigen binding site, composed of three complementarity determining regions (CDRs). Diversity in CDR amino acid composition across the B cell repertoire allows binding to a vast array of foreign and self-antigens. CDR3 encompasses the junction between variable (V), diversity (D), and joining (J) genes in the heavy chain (or V and J genes in the light chain), in which the random addition or deletion of nucleotides further increases diversity. Increased CDR3 length and positively charged amino acid residues have previously been associated with autoreactivity against nuclear antigens [65]. In addition to combinatorial and junctional diversity, somatic hypermutation further increases BCR diversity through random nucleotide mutations, initiated by activation-induced cytidine deaminase (AID), which can promote affinity maturation. Affinity maturation is a process by which B cells expressing BCR mutations that increase antigen binding strength compete more effectively for T cell help and outcompete lower-affinity B cell clones, which die due to neglect. Somatic hypermutation can also drive clonal redemption in which autoreactive B cells mutate away from self-reactivity and toward foreign antigen reactivity [110].

The role of somatic hypermutation in T1D development is unclear. One group reported that AID-deficient NOD mice exhibit increased B cell frequencies, enhanced T–B interactions, and develop accelerated T1D [40]. In contrast, another group showed that genetic disruption of AID or inhibition of RAD51-mediated DNA repair protects NOD mice from T1D through the expansion of regulatory B lymphocytes [41]. These discrepancies may be explained by differences in genetic methods used to generate these AID-deficient NOD mice. Tan et al. generated NOD.*Aicda*^−/−^ by backcrossing B6.*Aicda*^−/−^ mice to the NOD/Caj genetic background for more than 10 generations [40]. Ratiu et al. generated NOD.*Aicda*^−/−^ mice using CRISPR-Cas9 technology [41]. Both studies show increased B cells in AID-deficient NOD mice, but Ratiu et al. provide rationale that these B cells may have a regulatory impact on T cell function [40,41].

Limited studies exist which probe the role of somatic hypermutation and autoantigen affinity maturation in murine and human T1D. Schroer et al. immunized BALB/c mice with human and bovine foreign insulins and used hybridoma technology to identify eighteen anti-insulin IgG monoclonal antibodies (mAbs), the secreted form of the BCR. These mAbs ranged in affinity from 1 × 10^6^–7 × 10^8^ M^−1^ and included mAb125 with an affinity of ~3 × 10^8^ M^−1^ [111]. Thomas et al. used site-directed mutagenesis to show that the variable heavy-chain region of mAb125 (VH125) contains two amino acid replacements in the heavy-chain CDR2 that are necessary for insulin binding [112]. The germline correlate of VH125, VH281, has no measurable insulin binding when expressed as a soluble antibody [112]. Thomas and colleagues introduced either VH125 or VH281 as IgM-restricted BCR transgenes that paired with endogenous light chains to produce a semi-polyclonal repertoire in which 1–2% (VH125Tg) or 0% (VH281Tg) of mature B cells bind insulin [34]. VH125Tg.NOD mice showed increased T1D incidence that arose ~one month sooner than is typically observed in WT.NOD mice, whereas VH281Tg.NOD mice were protected from diabetes [34]. Thus, in this case, affinity maturation promoted T1D disease pathogenesis, but this affinity maturation was elicited by immunization to a foreign antigen, as opposed to spontaneous autoimmunity. Anti-peripherin B cell hybridomas were generated from islet-infiltrating B cells. BCR sequence evaluation identified both germline and mutated BCRs, but affinity was not measured [113]. Therefore, no conclusions can be made regarding the role of affinity maturation in this model. One of the germline BCRs identified by Carillo et al. was used to generate the NOD-PerIg mouse [38]. Diabetes development in this model suggests that germline, autoreactive B cells can promote T1D pathogenesis.

B cells isolated from distinct pancreatic islets in VH125Tg.NOD mice shared CDR replacement mutations [114]. Insulin-binding splenic B cells from prediabetic VH125Tg.NOD mice were biased toward Vκ4-74 and Vκ4-57 light-chain usage [115]. Investigation into a particular light chain, Vκ4-57-1, which pairs with VH125 to form an insulin-binding BCR in NOD mice, showed that whereas 28% of Vκ4-57-1 light chains expressed mutations in spleen isolates, 47% of Vκ4-57-1 isolates from the pancreas or pancreatic draining lymph nodes had undergone mutation [115]. Recombinant expression of antibodies using the same Vκ4-74 light-chain gene but derived from diabetes-prone VH125Tg.NOD or diabetes-resistant VH125Tg.C57BL/6-H2g7 mice had comparable affinities, but when the Vκ4-57-containing antibodies were compared, the affinity from the disease resistant mouse was lower compared to its diabetes-prone counterpart [116]. It is important to note that numerous Vκ genes were found to be polymorphic in NOD mice relative to the C57BL/6 strain [117], and that several of these polymorphisms (present in CDRs) were confirmed to enhance murine insulin (self-antigen) recognition in the NOD strain compared to C57BL/6 mice expressing the same VH125 transgene, further obscuring the role of BCR somatic hypermutation in T1D development [115].

Somatic hypermutation canonically occurs in transient structures in secondary lymphoid tissues, called germinal centers. The original anti-insulin BCR transgenic models were IgM restricted and expressed heavy- and light-chain transgenes that were randomly integrated into the genome. Site-directed models were subsequently developed in which these anti-insulin BCR transgenes were introduced at the physiologic IgH and Igk locus, VH125^SD^ [31,118] and Vκ125^SD^ [36], respectively, which enabled B cells to undergo isotype switch, somatic hypermutation, and receptor editing. Anti-insulin B cells can spontaneously adopt a germinal center phenotype and undergo limited class switching in NOD.VH125^SD^ mice in vivo [35], which is dramatically enhanced by the presence of anti-insulin 8F10 T cells following co-transfer into *Rag1*-deficient NOD recipients [46]. In contrast to these studies indicating T1D dependence on germinal center formation, ~50% of NOD.*SAP*-deficient mice develop diabetes, despite showing a strong, albeit incomplete, reduction in germinal center B cells [119].

BCR mutations in non-transgenic NOD mice have also been identified. One anti-insulin antibody generated from the spleens of NOD mice with recent-onset diabetes harbored three amino acid replacements in the heavy chain and two amino acid replacements in the light chain of the BCR compared to other mouse strains, but the lack of corresponding sequences in the NOD germline at the time limited conclusions regarding somatic hypermutation in the natural autoimmune response in NOD mice [120]. Hybridoma generation from spleens of naive NOD mice identified anti-insulin B cells that exhibited low affinity for insulin in solution (IC_50_ > 50 μM) and were polyreactive to environmental antigens and other autoantigens [121]. Analysis of the heavy- and light-chain variable region genes in six of these insulin-binding mAbs showed that these V gene segments exhibited little to no mutation and were used by autoantibodies in other autoimmune disease mouse models [121]. These findings in the transgenic and wildtype NOD models together suggest that somatic hypermutation is not required for insulin recognition in some cases but may be needed to reach threshold affinity for insulin binding in other cases. Koehli et al. used a transgenic mouse model, which allowed for the expression of membrane-bound and secreted ovalbumin (OVA) of varying affinities in the pancreatic islet beta cells, to measure optimal OT-1 OVA-specific CD8+ T cell receptor autoantigen affinity for inducing autoimmune diabetes. They identified the highest risk of developing autoimmune diabetes to be just above the negative selection affinity threshold, such that T cell negative selection is leaky and TCR affinity is sufficient to confer autoimmune pathology [122]. We hypothesize that a similar affinity threshold may promote autoreactive B cells to escape tolerance in T1D while limiting the development of high-affinity responses, such as in SARS-CoV-2 vaccination responses (K_D_~1 × 10^−9^–20 × 10^−9^ M) [123]. Future studies are needed to formally address this question.

In humans, analysis of five anti-insulin B cell clones isolated from a T1D patient treated with exogenous insulin showed amino acid mutations in the variable heavy-chain genes of all five clones [124]. Germline reversion of three amino acid mutations in the heavy chain and six in the light chain of an anti-insulin mAb derived from an insulin-treated T1D patient led to preserved, insulin-specific binding, but with decreased affinity compared to the native, mutated mAb [125]. A single amino acid mutation in the CDR2 of the heavy chain was responsible for the increased affinity of this mutated anti-insulin BCR [125]. This suggests that insulin has the potential to drive germline, anti-insulin B cells through affinity maturation [125], but it is unclear whether the mutations arose via a natural autoimmune response or via the foreign immune response to repeated human insulin injection known to occur in diabetic patients [126]. Thus, studies of insulin-reactive BCRs isolated from insulin-therapy-naïve individuals will be necessary to formally address the role of somatic hypermutation in T1D pathogenesis. Analysis of seven human monoclonal anti-GAD65 IgG autoantibodies generated from two patients with newly diagnosed T1D showed an increased frequency of replacement versus silent mutations in antibodies that showed medium-to-high affinity for GAD65 [127].

Only a few studies have investigated changes in autoantibody affinity for autoantigens with disease progression. The BABYDIAB study prospectively followed children of parents with T1D from birth. Insulin autoantibody affinity remained relatively stable from seroconversion to next follow-up visit, with a median of 6.5 years and a range of 9 months to 12.5 years [128]. Another study followed Finnish children with HLA-conferred diabetes risk from birth. A trend toward increased sera autoantibody affinity for insulin was observed only in those that exhibited low insulin affinity at initial seroconversion and eventually progressed to clinical T1D [129]. Studies in mice identified a potential disconnect between B cell pathogenic function and autoantibody production [31,76]. Thus, these polyclonal human studies address antibody response maturation but do not give clear insight into BCR evolution with disease progression; therefore, longitudinal studies of islet-reactive B cells (and the BCRs they express) are necessary to clarify whether BCR autoantigen affinity correlates with disease progression.

## 6. B-Cell-Targeted Immunotherapy in Human T1D

B cell depletion therapy is used in rheumatic disease management (e.g., systemic lupus erythematosus, Sjogren’s syndrome, and rheumatoid arthritis), in which rituximab is typically re-administered if/when relapse is observed or anticipated [130,131,132]. Adult patients tolerate rituximab, an anti-CD20 monoclonal antibody (mAb), relatively well, with upper respiratory tract infection, hypertension, nausea, and fatigue being the chief side effects reported in two rheumatic disease clinical trials [133,134]. Given the predicted importance of B lymphocytes in driving T1D in mouse studies, rituximab efficacy in T1D was tested. In a phase-2 clinical trial, a single course of rituximab treatment temporarily depleted B lymphocytes and preserved beta-cell function in new-onset T1D individuals, but by 18 months post-treatment, B lymphocyte counts had recovered to baseline (as expected for a single course of drug) and T1D ultimately progressed (Table 2) [135,136]. By 30 months post-treatment, C-peptide area under the curve, insulin dose, and HbA1c were no longer significantly different between the rituximab-treated and placebo groups [135]. Serum IgM levels were decreased up to two years post-treatment, while IgG levels were not impacted [135]. IAA decreased by 75% at 6 months, while GAD65, IA2A, and ZnT8A remained relatively stable after treatment [137]. Chamberlain et al. reported that rituximab treatment did not alter the frequency of autoreactive and polyreactive B cells in T1D patients and proposed this was due to the accumulation of newly generated clones that continue to escape immune tolerance after rituximab treatment cessation [138]. Herold et al. observed enhanced T cell proliferation in response to diabetes-associated environmental, islet, and neuronal antigens in rituximab-treated C-peptide responders [139]. Proposed explanations for this counterintuitive observation include altered T cell trafficking or the regulatory function of the T cells being assayed [139]. Herold et al. postulate that these regulatory T cells may be induced by IL-10 producing B cells that repopulate after rituximab cessation [139].

Unsurprisingly, rituximab treatment depleted protective antibody responses to vaccination against a neoantigen bacteriophage phiX174 administered to T1D individuals during the time of B cell depletion [158]. Antibody response to hepatitis A, tetanus, and diphtheria vaccination during the time of B cell recovery reached levels of clinical response but was still impaired compared to placebo-treated T1D individuals [158], highlighting concerns related to dampened infection and vaccination responses. Such a side effect would be particularly concerning in children, in whom immune memory to many commonly encountered pathogens is still forming.

In addition to concerns related to diminished protective immunity in children with T1D, NOD mouse studies show that anti-CD20 antibody is unable to prevent late-stage diabetes (after insulin autoantibodies are present), due in part to the downregulation of B cell CD20 expression in the pancreatic islets [159]. A diminished frequency of IL-10-producing regulatory B cells has been associated with T1D in humans [160]. Rituximab treatment of NOD mice led to the depletion of IL-10-producing regulatory B cells, which play a critical role in regulating self-tolerance [161,162]. Taken together, these findings highlight the promise of B cell-directed therapy in T1D but suggest a need for the sustained disruption of autoreactive B cell function. Global B cell depletion carries unacceptable risk for pediatric individuals and likely diminishes protective responses by regulatory B cells, pointing to a need for more selective therapies that spare protective immune responses.

## 7. Antigen-Specific Therapy in T1D

Antigen-specific therapy is an attractive strategy to prevent T1D onset and progression while preserving protective immune responses. Many antigen-specific therapeutic strategies have been tested, as summarized in Table 2. Strategies to promote immune tolerance against T1D autoantigens include GAD peptide immunization, oral insulin administration, and proinsulin-encoding plasmid DNA immunization [152], as reviewed by Zhang et al. [149]. In a study of autoantibody-positive relatives of patients with T1D, oral insulin compared to a placebo did not delay or prevent the development of T1D over 2.7 years [150]. In a study of young, genetically at-risk children, immune response to oral insulin therapy, including insulin autoantibody levels and CD4+ T cell responses, were evaluated and no differences were observed compared to placebo controls [151]. An association between oral insulin therapy and insulin antibody response was observed in children with the susceptible INS AA genotype [151]; therefore future oral insulin administration studies may benefit from immune-based patient stratification.

Modified insulin molecules have also been analyzed for therapeutic potential. A monomeric insulin–CD22L conjugate, which targets both insulin-reactive BCRs and the inhibitory receptor CD22, reduced B cell activation in response to simulated T cell help and reduced pathogenic anti-insulin B cells from 125Tg^SD^.B6 mice in vitro [156]. A recent study demonstrated diabetes protection in the highly aggressive VH125Tg.NOD model when mice were treated with the insulin–Fc fusion drug, AKS-107 [157]. Key features of AKS-107 include modifications to the insulin moiety that (1) prevent signaling via the insulin receptor and (2) support the induction of immune tolerance in anti-insulin T cells. As such, AKS-107 selectively eliminated anti-insulin B lymphocytes and supported durable protection against T1D in VH125Tg.NOD mice [157]. Soluble antigen arrays consist of multiple peptides conjugated onto small hyaluronic acid chains to mimic high-avidity interactions and promote B cell anergy [153]. Soluble antigen arrays that carried a mixture of two autoantigen peptides stimulated non-overlapping endogenous T cell populations, induced the expression of tolerance markers, and blocked T1D development in NOD mice adoptively transferred with T cells specific for these two peptides [154]. This method prevented the anaphylaxis observed in response to free peptide administration [154].

Selective elimination of insulin-reactive B lymphocytes through the administration of an anti-insulin mAb prevented T1D in NOD mice [80]. The efficacy was attributed to the Fc-mediated elimination of anti-insulin B lymphocytes and potential reinforcement of central tolerance in the bone marrow through increased insulin antigen:BCR crosslinking, which enhanced receptor editing [109]. The intraperitoneal injection of purified IgM isolated from Swiss Webster mice into VH125^SD^.NOD mice resulted in a complete loss of detectable insulin-binding B cells in the spleen [155]. A similar strategy currently being studied in NOD mice is Pentaglobin treatment, a human immunoglobulin preparation enriched in IgM, which led to the expansion of thymic B cells and regulatory T cells (Tregs) and short-term reversal of T1D in ~78% of mice enrolled in the study [163]. Translation of these antigen-specific therapies from mouse to human may support the development of successful non-immunosuppressive T1D treatments in the future.

## 8. T-Cell-Targeted Therapies in T1D

T-cell-targeted therapies have also been investigated for the treatment of T1D (Table 2) [164]. Teplizumab, an anti-CD3 antibody, was shown to preserve C-peptide responses in patients with new-onset T1D and was recently FDA approved as the first T1D therapy to prevent progression from stage 2 to stage 3 of the disease, but it shows heterogenous responses [140,141,142,143]. The mechanism of action is not fully understood, but data from two randomized clinical studies of teplizumab in new- and recent-onset T1D patients revealed that clinical responders showed a significant reduction in circulating CD4+ effector memory T cells and an increase in the frequency and absolute number of CD8+ central memory T cells [142].

Abatacept, a CTLA4Ig, has also been tested in clinical trials for T1D treatment in new-onset T1D patients [144]. CTLA4Ig is a soluble CD28 inhibitory homolog that binds CD80/CD86 on APCs, including B cells, to prevent necessary T cell priming and helper functions [165]. As observed in rituximab and teplizumab studies [135,136,143], abatacept treatment resulted in a significant but transient delay in C-peptide loss with a heterogenous response [144]. To investigate the mechanisms involved in the response to abatacept, Linsley et al. performed modular gene expression analysis, flow cytometric B cell subset analysis, and insulin autoantibody measurements of abatacept-treated responder versus non-responder subjects. Results showed increased activated B cell gene expression and B cell frequency in non-responders compared to responders [145]. Edner et al. showed that lower frequencies of ICOS+ Tfh cells at baseline were associated with response to abatacept treatment [146]. These findings suggest that targeting the B–T axis may be a beneficial strategy to target both pathogenic B and T cell responses.

Combined B and T cell-targeted therapy increased efficacy in a clinical trial investigating Treg and rituximab combination therapy, as compared to Treg therapy or insulin therapy alone [147]. A recent clinical trial showed that combination therapy with AG019, a bacteria genetically modified to express human proinsulin and human IL-10, and teplizumab led to stabilization or improvement in all measured metabolic variables up to 12 months and significantly increased exhausted CD8+ T cells at 6 months compared to AG019 monotherapy or placebo controls [148]. It should be noted that rituximab alone or teplizumab alone controls were not included in these studies to confirm that the increased efficacy was not solely due to rituximab or teplizumab treatment, respectively [147,148].

## 9. Conclusions

Although beta-cell destruction in T1D is T cell-mediated, B cells play a crucial role in T1D pathogenesis through autoantigen presentation. As reviewed here, the impact of BCR somatic hypermutation and affinity maturation on T1D-associated autoantigen recognition is still unclear with respect to the natural evolution of autoimmune responses in the pre-diabetic interval. Alterations in B cell signaling impact the development and expansion of islet-reactive B cells in T1D, highlighting additional targets for T1D prevention. B cell-targeted therapies showed some promise in clinical trials, but limitations included non-durable and heterogenous responses and concern over side effects arising from undesirably broad immunosuppression. The role for germinal center entry, somatic hypermutation, and affinity maturation in supporting T1D is still not entirely clear. Future studies to address these aspects of pathologic B cell activity in T1D could lead to the identification of novel T1D biomarkers and drugs that could improve clinical trial development, the evaluation of new therapies, and disease management in the clinic.

## Figures and Tables

**Table 1:** Examples of NOD mouse models used to dissect B cell functions in T1D.

Name	Description/Major Findings	T1D?	Reference(s)
NOD	- Classic spontaneous T1D model.	Develops	[16]
NOD.Igμ	- B-cell deficiency; - B cells present antigen to confer T1D.	Protected	[7,32]
NOD BCIID	- MHC class II I-Ag7 deficiency confined to B cells.	Protected	[33]
NOD.125Tg	- Anti-insulin heavy- and light-chain B cell receptor (BCR) transgenes; - IgM restricted; - >95% of developing and peripheral B cells bind insulin; - B cells are anergic; - Impaired insulin autoantibody production.	Develops	[30]
NOD.VH125Tg	- Anti-insulin heavy chain transgene pairs with endogenous light chains; - IgM restricted; - 1–2% of developing and peripheral B cells bind insulin; - Impaired insulin autoantibody production.	Accelerated	[34]
NOD.VH281Tg	- Heavy chain transgene–VH125 derivative lacking two key amino mutations necessary for insulin recognition; pairs with endogenous light chains; - 0% of B cells bind insulin.	Protected	[34]
NOD.VH125^SD^	- Anti-insulin BCR transgene targeted to IgH locus; - Isotype switch and somatic hypermutation possible; - Anti-insulin B cells enter germinal centers but are blocked from antibody production.	Accelerated	[31,35]
NOD.VH125Tg.VΚ125^SD^	- Anti-insulin heavy-chain BCR transgene pairs with endogenous light chains; - Anti-insulin BCR transgene targeted to Igκ locus; - IgM-restricted; - ~50% of peripheral B cells bind insulin; - Receptor editing reduced compared to non-autoimmune mice.	Not reported	[36]
NOD.*Btk*^−/−^	- Bruton’s tyrosine kinase (BTK) deficient (disrupted BCR signaling); - Decreased follicular/marginal zone B cells.	Protected	[37]
NOD.VH125Tg. *Btk*^−/−^	- Anti-insulin heavy chain BCR transgene pairs with endogenous light chains; - IgM restricted; - 1–2% of developing and peripheral B cells bind insulin (numbers reduced); - BTK deficient.	Develops	[37]
NOD.PerIg	- Anti-peripherin heavy- and light-chain BCR transgene.	Accelerated	[38]
NOD.scid.PerIg	- Anti-peripherin heavy- and light-chain BCR transgene; - T-cell deficiency.	Accelerated (with T cell transfer)	[38]
NOD.IgHEL	- Anti-hen egg lysozyme (HEL) heavy- and light-chain BCR transgene.	Delayed	[8]
NOD.IgHEL.Igμ	- Anti-HEL heavy- and light-chain BCR transgene; - Completely lacking B cells able to bind beta-cell antigens (no endogenous BCR).	Protected	[8]
NOD.SCID	- NOD mice that lack B and T cells.	Protected	[38]
NOD.116C	- Unknown beta-cell antigen-specific heavy- and light-chain BCR transgene; - B cells are anergic.	Protected	[39]
NOD.*Aicda*^−/−^	- AID deficient (cannot undergo somatic hypermutation or class switching).	Accelerated	[40]
NOD.*Aicda*^−/−^	- AID deficient (cannot undergo somatic hypermutation or class switching); - Expanded regulatory B cells.	Protected	[41]
NOD.*Tlr7*^−/−^	- TLR7 deficient.	Protected	[42]

**Table 2:** Experimental immunotherapies that impact B and T cell function and diabetes outcomes.

Therapy	Target	T1D Outcomes	Reference(s)
In Human			
Rituximab	CD20+ B lymphocytes	Temporary preservation of beta-cell function in new-onset T1D individuals	[135,136,137,138,139]
Teplizumab	CD3+ T lymphocytes	Preservation of beta-cell function in new-onset T1D individuals over 2-year follow-up period, prevents progression from stage 2 to stage 3	[140,141,142,143]
Abatacept	CD80/86+ APCs	Preservation of beta-cell function in recent-onset T1D over 2-year treatment period	[144,145,146]
Treg and rituximab combination therapy	Tregs suppress immune response, rituximab targets CD20+ B lymphocytes	Preservation of beta-cell function in pediatric new-onset T1D individuals over 2-year follow-up period	[147]
AG019 bacteria and teplizumab combination therapy	AG019 bacteria genetically modified to express human proinsulin and IL-10 to promote tolerance, teplizumab targets T lymphocytes	Preservation of beta-cell function in new-onset T1D individuals over 12-month follow-up period	[148]
GAD peptide immunization	GAD-specific B and T lymphocytes	Variable impact on beta-cell function	[149]
Oral insulin	Insulin-specific B and T lymphocytes	Variable immune responses	[149,150,151]
Proinsulin-encoding plasmid DNA immunization	(Pro)insulin-specific T lymphocytes	Preservation of beta-cell function in adult T1D individuals over 15-week follow-up period	[152]
**In Mouse**			
mAb123	Insulin-bound B lymphocytes	Protects from T1D	[80]
Soluble antigen array	Autoantigen-specific B and T lymphocytes	Protects from T1D	[153,154]
Healthy polyclonal IgM	Insulin-binding B lymphocytes	Reverses T1D	[155]
Insulin-CD22L conjugate	Insulin-binding B lymphocyte	Reduced anti-insulin B cell proliferation with anti-CD40 stimulation in vitro	[156]
AKS-107	Insulin-binding B lymphocytes	Protects from T1D	[157]

## Data Availability

Not applicable.

## Abbreviations

Activation-induced cytidine deaminase (AID), antigen-presenting cells (APCs), B-cell-activating factor (BAFF), BAFF receptor (BAFFR), B-cell receptor (BCR), Bruton’s tyrosine kinase (BTK), complementarity determining regions (CDRs), diversity (D), glutamic acid decarboxylase 65 (GAD65), hemoglobin A1c (HbA1c), insulin autoantibody (IAA), tyrosine phosphatase-related islet antigen-2 (IA-2), islet cell autoantigen (ICA), immunoreceptor tyrosine-based activation motifs (ITAMs), joining (J), Juvenile Diabetes Research Foundation (JDRF), major histocompatibility complex (MHC), non-obese diabetic (NOD), Network for Pancreatic Organ Donors with Diabetes (nPOD), ovalbumin (OVA), phosphatidylinositol-specific phospholipase Cγ2 (PLCγ2), type 1 diabetes (T1D), toll-like receptors (TLRs), regulatory T cells (Tregs), variable (V), zinc transporter 8 (ZnT8).
